# Comparison of Alkaline/Oxidative and Hydrothermal Extraction of Wheat Bran Arabinoxylans

**DOI:** 10.3390/foods10040826

**Published:** 2021-04-10

**Authors:** Marcus Schmidt, Berthold Wiege, Jürgen Hollmann

**Affiliations:** Max Rubner-Institut, Research Institute of Nutrition and Food, Institute of Safety and Quality of Cereals, Schützenberg 12, 32756 Detmold, Germany; Berthold.Wiege@mri.bund.de (B.W.); jhollman@mail.uni-paderborn.de (J.H.)

**Keywords:** wheat bran, arabinoxylans, alkaline/hydrogen peroxide extraction, hydrothermal extraction

## Abstract

The bran accounts for approximately 25% of the wheat kernel but is currently only a by-product, used as animal feed. However, due to its high arabinoxylan content it could be a valuable raw material for food production. Arabinoxylans cannot be digested in the human intestine but are intensely studied for their health-beneficial properties. These include glycemic control by formation of a highly viscous gel in the intestine, and hence delaying starch digestion, alongside an increase in short chain fatty acids. To apply sufficient amounts of arabinoxylan for health-beneficial effects, extraction and concentration is required. Alkaline/oxidative conditions are commonly used, but for potential food applications more cost-efficient methods, without hazardous chemicals, are required. Therefore, this study aimed to optimize the conditions for hydrothermal extraction (extraction time and temperature) at laboratory-scale and to compare the results to an established alkaline/oxidative method. The resulting extracts were characterized for yield, purity, arabinoxylan molecular mass, arabinose/xylose ratio, and viscosity to evaluate the quality of the method. For the hydrothermal extraction, an extraction time of 1 h at 160 °C and 6.5 bar gave the best results. However, even these optimized conditions resulted in lower extract purity and severely degraded arabinoxylans. Although further optimization of the hydrothermal process is required, the present work builds an important foundation for the development of an industrial hydrothermal extraction method.

## 1. Introduction

After rice and maize, wheat is one of the most important cereals worldwide. Together these three crops comprise over 90% of the global cereal production. Out of the more than 765 million tons of annually produced wheat, over 69% are used for human consumption [1,2]. However, a number of valuable components are removed as by-products during the milling process. These components include the bran, germ, and parts of the endosperm [2]. Since wheat bran accounts for approximately 25% of the whole kernel, the annually removed bio-mass is estimated at 150 million tons, which is primarily used as animal feed [2,3].

In addition to the large volume of readily available wheat bran, a number of studies have linked it to several health-beneficial properties. The topic was thoroughly reviewed by Stevenson et al. [4]. On the downside, however, the technological performance of wheat bran is generally poor, making the direct application in food products difficult. Despite the increasing efforts to utilize wheat bran more frequently, usages are still limited, and further research is required to allow a universal application [5]. A promising approach is the extraction and concentration of selected bran constituents with proven health benefits. This could evolve wheat bran from a by-product with health functions, into a material with high industrial applicability, along with health functions.

Wheat bran mainly consists of non-starch polysaccharides, but also starch, protein, and some minor constituents [6]. The group of non-starch polysaccharides is comprised of a number of different components, including cellulose, lignin, and arabinoxylan (AX). Due to its high concentration in the bran (approximately 25%) and its proven health-endorsing properties, AX is of particular interest. AX was studied for the control of the glycemic index, due to delayed starch digestion and glucose absorption in the human intestine. Its contribution to the control of the blood glucose rise after food consumption is also recognized by the European Food Safety Authority (EFSA) [7]. In addition, AX was shown to promote the production of short chain fatty acids (SCFA) in the human intestine and to exhibit a preventative effect against colon cancer [8]. On a molecular level, AX is comprised of linear chains of *β*-(1,4)-linked d-xylopyranosyl residues. These chains are substituted with l-arabinofuranose side chains on the *O*-2 and/or *O*-3 positions [9]. Furthermore, different organic acids, such a glucuronic or ferulic acid can be attached to the arabinose side chains.

There are a number of different extraction methods reported for AX, including chemical, hydrothermal, enzymatic, and mechanical treatments [10]. However, the requirements for an extract with possible food applications are manifold. The most important criteria include, high yield and extract purity, the absence of hazardous chemicals, cost-efficiency, and the preservation of the nutraceutical properties.

Using a simple aqueous extraction, only a relatively small percentage of the total AX can be obtained. Early studies on aqueous extraction procedures for cereal AX reported yields below 1% [11,12]. The reason behind this is the covalent and non-covalent binding to the cell-wall components of the bran. Based on such results, Izydorczyk and Dexter [13] proposed that aqueous extraction at temperatures below 100 °C cannot break the links between AX and the cell-walls. One popular approach to break these links is extraction with alkaline/hydrogen peroxide solution. However, alkaline extractions usually result in de-acetylation and de-esterification of AX, and the resulting extracts have to be neutralized and desalted before they can be used in food products [14]. Furthermore, this process requires expensive chemicals, which leave environmentally damaging ions, such as Ba^2+^ [10]. Yet another approach is the use of supercritical water at temperatures above 100 °C under high pressure (hydrothermal) to release the AX from the attached cell-wall components. The improved solubility of AX in water at temperatures of 180 °C or above was reported by Zhang, Smith, and Li [10]. However, aqueous extractions usually still result in lower yields and purities compared to chemical extractions. In addition, the extracted AX experiences some substantial quality deterioration in the process [10]. The most crucial quality parameters include the molar mass (MW) and arabinose/xylose (A/X) ratio, among others. It was reported, that changes in MW and/or A/X can drastically alter, and possibly compromise, the health-endorsing properties of AX [15].

The aim of this study was the production of a high-quality AX extract for possible food applications, using a hydrothermal method. Therefore, the ideal process parameters for a hydrothermal extraction were determined at laboratory scale. A product obtained by an established alkaline/hydrogen peroxide extraction at pilot-scale was used as a reference point to evaluate the hydrothermal extracts. The quality of each method was judged based on the yield and purity, as well as the MW, A/X, and dynamic viscosity of the products. Therefore, this study provides important information on the path to an efficient, cost-effective, and safe extraction of AX for use as a functional food ingredient.

## 2. Materials and Methods

### 2.1. Materials and Raw Material Characterization

Industrial ethanol (96%), denatured with 2% petroleum ether was purchased from Alkohol Handelskontor (Lippstadt, Germany). Unless stated otherwise, all used chemicals were purchased from Merck KGaA (Darmstadt, Germany) and at least of reagent grade purity. The alcalase (2.4 L, food grade) was purchased from Novozymes A/S (Bagsvaerd, Denmark). Monosaccharide standards for arabinose and xylose (purity > 95%) were purchased from Sigma Aldrich GmbH (Taufkirchen, Germany).

Wheat bran was purchased from Kampffmeyer Mühlen GmbH (Hamburg, Germany). The raw material was characterized by determining moisture, ash, lipid, and protein content according to ICC (International Association for Cereal Science and Technology) standard methods No. 109/1 [16], 104/1 [17], 136 [18], and 105/2 [19]. Starch content was determined according to the Ewers method (ISO 10 520:1997) [20]. The contents of total, soluble, and insoluble fiber were determined according to the AOAC (Association of Official Analytical Chemists) method 991.43 [21], using an enzymatic test kit from Megazyme (Bray, Ireland). The amount of arabinoxylan present in the bran was calculated from the arabinose and xylose contents, as described in Section 2.6.

### 2.2. Alkaline/Hydrogen Peroxide Extraction

**Figure 1:**
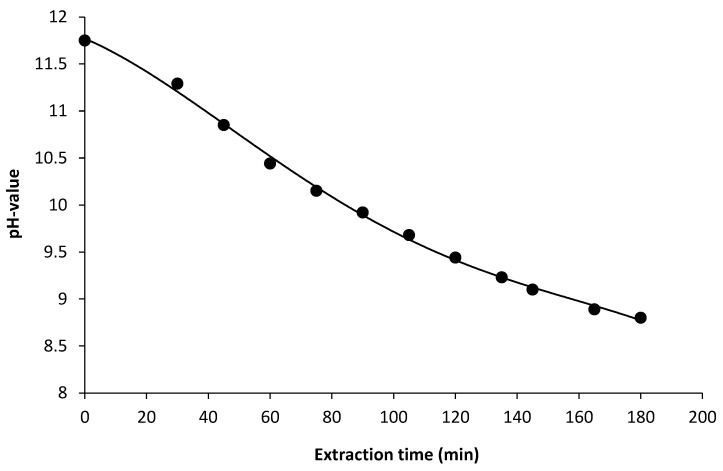
Changes in pH-value of the extraction slurry as a function of the extraction time.

For alkaline/hydrogen peroxide extraction of AX, the method of Hollmann and Lindhauer [22] was adapted with some modifications. In brief, 3.46 kg of wheat bran were suspended in 34.6 kg of water and stirred for 30 min. Subsequently, starch and other cold-water soluble components were removed by wet sieving through a 250 µm curved screen. The sieve rejection (18.54 kg) was washed with deionized water and suspended in 17.07 kg of water to give a total of 33.90 kg of slurry, with 6.43% dry matter (DM). Then the suspension was heated to 80 °C and held at this temperature for 15 min. After cooling the slurry to 62 °C, 400 mL of 20% sodium hydroxide solution was added. This resulted in a total amount of 2.03 mol sodium hydroxide in the extraction slurry, with pH = 11.75. Simultaneously, 769 g of hydrogen peroxide solution (30%) was added to the mixture with a feed rate of 4.3 g/min. The extraction/hydrolysis was allowed to continue for 180 min under constant stirring and monitoring of pH (Figure 1).

After the extraction step, the sediment was separated from the supernatant by centrifugation, washed with deionized water, and centrifuged again. The combined supernatants (31.99 kg) were subjected to an alcalase-treatment, to prevent membrane fouling in the following filtration steps. Therefore, 50 mL of Alcalase 2.4 L were added to the supernatant and the mixture was stirred for 12 h at room temperature. The enzyme was inactivated by subsequent heating of the mixture to 100 °C for 10 min.

**Figure 2:**
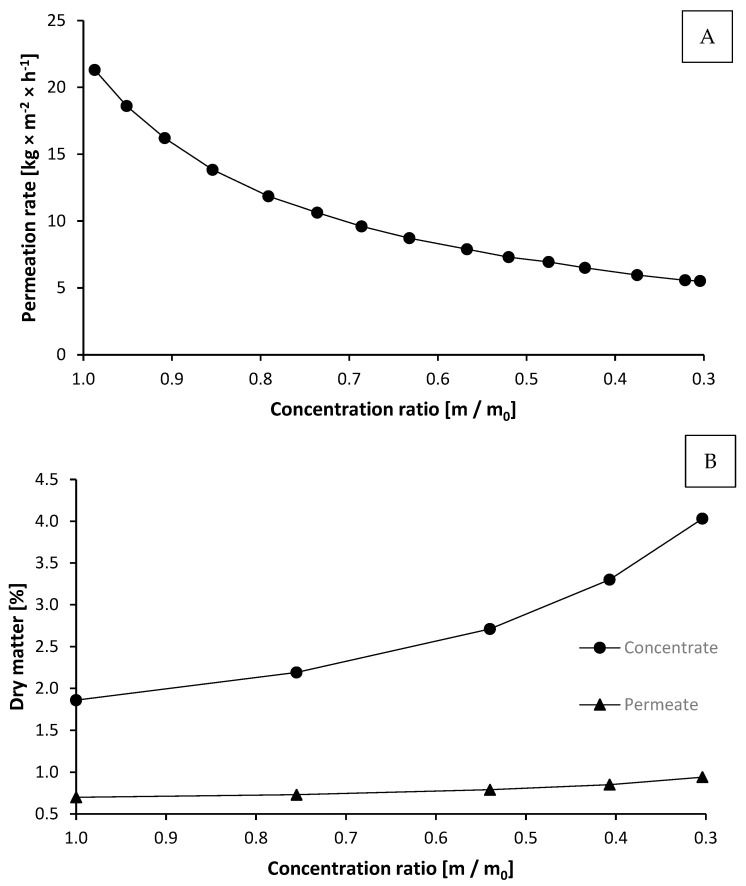
Development of the permeation rate (**A**) and dry matter (**B**) of concentrate and permeate as a function of the concentration ratio during ultrafiltration. Thereby, m is the current mass of the concentrate during filtration and m0 the mass of the concentrate at the beginning of the process (31.99 kg).

For concentration of the supernatants by ultrafiltration (UF) and the following purification by diafiltration (DF) a plate and frame process unit with an Alfa Laval Lab Stak^®^ M38H module (Alfa Laval Mid Europe GmbH, Glinde, Germany) was used. Fluoropolymer membranes ETNA 010A with a molecular cut off at MW = 10,000 g/mol were utilized for the concentration/separation of the arabinoxylans. The filtration was carried out using the following process parameters: Temperature = 39.7 °C, feed rate = 3600 kg/h, membrane area = 0.60 m^2^, pH = 8.32, inlet pressure p_in_ = 3.25 bar, and outlet pressure p_out_ = 2.75 bar. The permeation rate as a function of the concentration ratio (m/m_0_) is presented in Figure 2A. The permeation rate decreased rapidly from about 22 to 5.52 kg·m^−2^·h^−1^, where a concentration ratio of m/m_0_ = 0.304 was reached. This means the slurry with the arabinoxylan product was concentrated from 31.99 to 9.72 kg. The dry matter contents of the concentrate and permeate during UF, as function of the concentration ratio, are shown in Figure 2B.

During filtration, the dry matter of the concentrate increased from 1.86 to 4.03%, while the dry matter of the permeate increased only from 0.70 to 0.94%.

**Figure 3:**
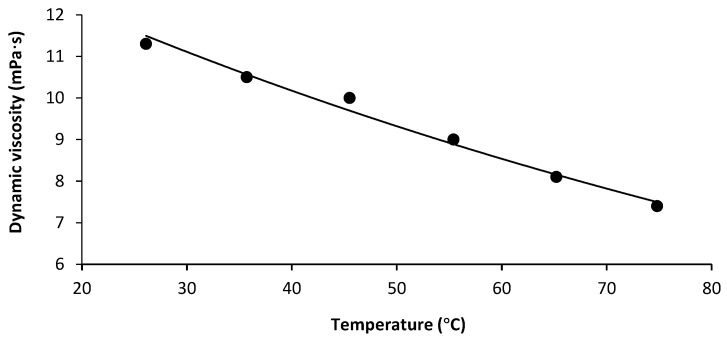
Dynamic viscosity of the ultrafiltration retentate (dry matter = 4.03%) as a function of measuring temperature, determined at a shear rate of 1872 s^−1^.

In order to clarify if further purification by diafiltration was possible, the dynamic viscosity of the retentate, obtained from the UF, was determined. Despite the high AX concentration, the viscosity of the concentrate (approximately 10 mPa·s) was low enough for diafiltration and potential technical applications in the relevant temperature range of 40–50 °C (Figure 3).

**Figure 4:**
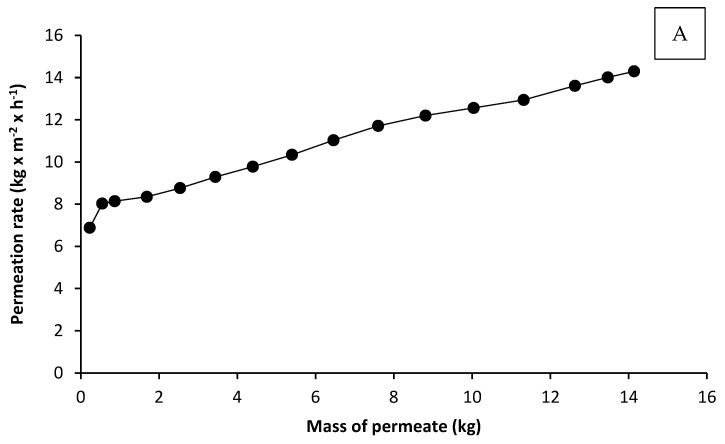

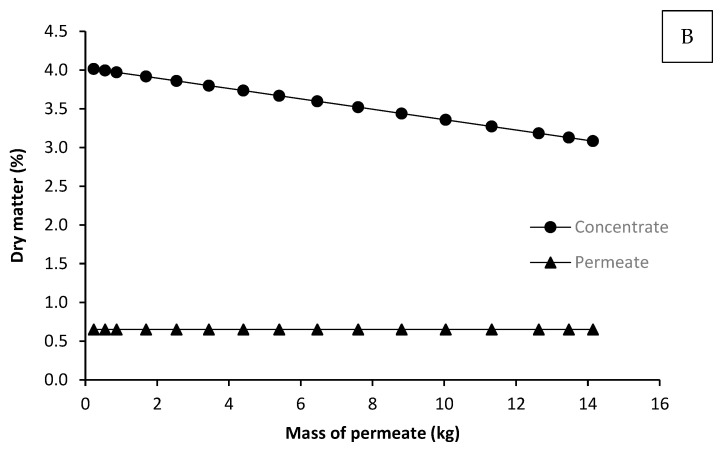
Permeation rate during diafiltration (**A**), and dry matter of the concentrate and permeate (**B**), as a function of the mass of the permeate. Diafiltration was carried out at constant mass of the concentrate (9.72 kg).

For further enrichment of AX, the UF concentrate was diafiltrated with 14.1 kg of deionized water at T = 55.2 °C and a constant mass of concentrate of 9.72 kg. During this process the permeation rate increased from 7 to 14 kg·m^−2^·h^−1^ (Figure 4A).

At the same time, the dry matter content of the concentrate was reduced from 4.03 to 3.08% (Figure 4B).

To precipitate the arabinoxylans from the concentrate, ethanol (96%) was added to a final mass ratio of 3.17/1 for ethanol/concentrate. The precipitated arabinoxylans were separated by centrifugation and dried. The AX content in the final product was determined, as described in Section 2.6, and used to calculate extraction yield and extract purity.

### 2.3. Hydrothermal Extraction

In order to identify the best conditions for hydrothermal extraction, a series of laboratory-scale extractions were carried out. Therefore, the wheat bran was first de-starched by wet sieving, as described in Section 2.2.

For the extraction of AX, 12.0 g (11.28 g DM) of de-starched wheat bran was suspended in 200 mL of water. The slurry was then subjected to hydrothermal aqueous extraction, in a stirred 400 mL autoclave using different extraction times (30 min, 60 min, and 120 min) and temperatures (22–163 °C) to determine the optimal parameters. At the highest extraction temperature (163 °C) a pressure of 6.5 bar was obtained. After extraction was completed, the reaction slurry was centrifuged, and the sediment was washed with distilled water and centrifuged again. From the combined supernatants AX were precipitated by the addition of ethanol (96%) at various mass-ratios between 1–5/1 for ethanol/water. The precipitate was recovered by filtration and dried at room temperature.

### 2.4. Determination of Molecular Weight

In order to determine the molecular weight distribution of the arabinoxylans, aqueous solutions containing 0.2% AX were prepared. To ensure complete dissolution of the samples, the mixtures were stirred for 30 min at 95 °C. After cooling to room temperature, the solutions were filtered (0.45 µm) into a GPC (Gel Permeation Chromatography) vial.

Molecular weight distribution of the isolated AX was determined by HPSEC using two ViscoGel GMPWXL Mixed Bed (7.8 × 300 mm) columns in series (Viscothek, Weingarten, Germany). The injection volume was set to 100 µL and the column temperature held consistently at 35 °C, using 0.5 mol/L sodium nitrate, containing 0.02% sodium azide, as eluent and operating at a flow rate of 0.6 mL/min. The average molecular weight and intrinsic viscosity were determined using a triple detection system, TDA 302, composed of a dual refractometer/viscometer detector in combination with a multi-angle laser light scattering detector (MALLS; Viscothek, Weingarten, Germany). For every sample, three independent determinations were carried out and the average curves determined. Molecular weights and viscosities were calculated using the calibration modules of Trisec software (Viscothek). The system was calibrated with pullulan standards and dn/dc = 0.147.

### 2.5. Arabinose/Xylose Ratio

The determination of the monosaccharides was based on the method previously published by Hollmann and Lindhauer [22], with some modifications. In brief, 20 ± 1 mg of sample was weighted accurately in a closed glass vessel and hydrolyzed at 105–110 °C for 2 h, using 2.0 mL sulfuric acid (1.0 mol/L). After cooling to room temperature, the hydrolysate was neutralized with sodium hydroxide (0.2 mol/L). The sulfate ions were precipitated by the addition of barium hydroxide (0.25 mol/L). The samples were centrifuged (2000× *g*, 20 °C, 10 min) and the supernatant was filtered (pore size 0.45 µm) into a HPLC sample vial.

The extracts were quantified by high-performance anion exchange chromatography with pulsed amperometric detection (HPAEC-PAD) on a DIONEX BioLC system (Dionex Corporation, Sunnyvale, CA, USA). The system contained a gradient pump GS50 and an electrochemical detector ED50. The separation was carried out with a DIONEX CarboPac PA1 analytical column (4 × 250 mm) equipped with a CarboPac PA1 guard column (4 × 50 mm). For the electrochemical detector, a reference Ag/AgCl electrode was used with a working gold electrode, applying a gold carbo quad waveform. The injection volume was 25 µL and analytes were eluted under isocratic conditions with 20 mM sodium hydroxide at a 1.0 mL/min flow rate and 20 °C within 25 min.

The system was calibrated with arabinose and xylose standards in distilled water. Calibration standards containing 2–50 mg/L arabinose or xylose were used. The calibration curve was obtained by plotting the mean peak area of 6 replicates against the respective concentration, resulting in correlation coefficients of 0.96 and 0.99 for linear regression. The limit of detection (LOD) and limit of quantification (LOQ) were calculated from the calibration curves using STATIST 2.0 software, based on the German DIN standard 32645 [23]. For arabinose and xylose, the LOD was calculated to 1.4 mg/L and 1.6 mg/L, and the LOQ was calculated to 4.8 mg/L and 5.6 mg/L, respectively. All analyses were conducted in triplicate, and values were required to be within a range of 2% from the mean value. The AX content of samples was calculated as 0.88 (mean% arabinose + mean% xylose).

### 2.6. Determination of Viscosity of Extracted AX

The viscosity of the AX extracts in aqueous solution was measured using a viscotester VT 550 with the measuring equipment MV 1 (Haake Meßtechnik GmbH & Co, Karlsruhe, Germany). The final extracts obtained from the alkaline/oxidative and hydrothermal extraction were dissolved in deionized water to final concentrations between 3.5% and 7.5% (based on dry matter) A circulating thermostat (F 25-MV, Julabo, Haake Meßtechnik GmbH & Co, Karlsruhe, Germany) filled with silicone oil was used to keep the measuring temperature constant in the range of 20 to 50 °C. All measurements were carried out at a shear rate of 179.6 s^−1^ to determine the dynamic viscosity. To characterize the AX, the dynamic viscosity was plotted against the solution concentration at different measuring temperatures.

## 3. Results and Discussion

### 3.1. Wheat Bran Characterization

Prior to the extraction, the wheat bran was subjected to compositional analysis. The results are summarized in Table 1.

The wheat bran used in this study had a dry matter (DM) of 86.7%. Approximately half of this dry matter (49.4%) was identified as dietary fiber. Further analysis of the fiber fraction showed that it was comprised of 96.6% insoluble fiber and 3.4% (water)soluble fiber. Alongside cellulose, lignin, and *β*-glucan, AX is a major dietary fiber component in wheat bran. Here, the AX content was determined as 24.5% in DM, which equals approximately 50% of the total dietary fiber. The remaining half of the bran dry matter was identified as protein (17.3% in DM), starch (18.5% in DM), minerals (6.1% in DM), and lipids (3.4% in DM).

The analytical results obtained from the wheat bran are in agreement with the values reported by Maes and Delcour [24], as well as Hollmann and Lindhauer [22]. The A/X ratio was determined to 0.6, which is similar to previously reported results for native [24] and de-starched [25] wheat bran. In particular, the high AX content highlights the potential of wheat bran as a source of health-beneficial dietary fiber. Considering its current use as animal feed, this study can provide a substantial valorization for this abundently available milling by-product.

On the other hand, the AX content is not sufficient to warrant the direct use of wheat bran in food products. Application of wheat bran, rich in cellulose and lignin, was shown to have substantial negative impact on bread quality and consumer acceptance [26,27]. In addition, the EFSA health-claim for AX can only be issued if AX-rich substrates (containing at least 60% AX) are used [7]. Hence, despite being a valuable source of AX, wheat bran requires further processing and refining before it can contribute to an AX-rich diet, and be advertised with the corresponding health-claim [7]. To this end, different procedures are examined and compared in the following sections.

### 3.2. Extraction of Arabinoxylans

AX was extracted from de-starched wheat bran at pilot-scale, using alkaline/hydrogen peroxide. This method, used as a reference, was based on the procedure previously described by Hollmann and Lindhauer [22]. The authors reported high extract yield and purity for this method. In comparison, the hydrothermal method described here was established with the aim to obtain results of similar quality but without the use of expensive and potentially hazardous chemicals. In order to determine the ideal process parameters for the hydrothermal extraction, a range of selected crucial process parameters were investigated at laboratory-scale.

**Figure 5:**
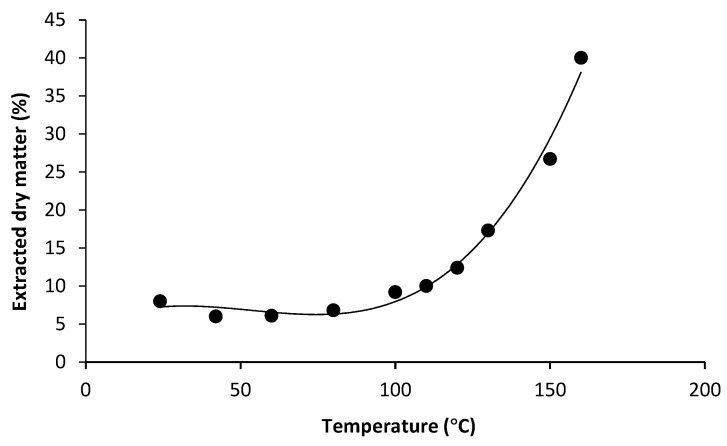
Extracted dry matter as a function of the extraction temperature, obtained after 2 h of hydrothermal extraction at laboratory-scale.

Among the most important parameters for an efficient extraction is the temperature [28]. The relation between the extraction temperature and the extracted dry matter is shown in Figure 5.

From the results, it is clearly visible that extraction temperatures between 20 °C and 80 °C resulted in very low extracted dry matter, ranging between 6–8%. Upon reaching an extraction temperature of 100 °C, the efficiency started to increase rapidly. The highest extracted dry matter (40%) was determined for the highest temperature (160 °C).

From the results presented in Figure 5, one could expect an even higher rate of extraction from a temperature above 160 °C. However, it was previously demonstrated by Sanchez-Bastardo, et al. [29] that temperatures above 160 °C compromise the quality of the AX extract. The authors reported an increased total AX yield for a temperature of 180 °C. However, this was due to an increase in monomeric arabinose and xylose, released by autocatalysis of the AX polymer. As a consequence, the extract purity and yield for oligo- and polymeric AX, as well as the MW of the extracted AX were substantially reduced compared to 160 °C. Similar results with regards to the extraction temperature were reported by Aguedo et al. [30]. According to the results obtained in this preliminary trial, the highest temperatures included in the study (150 °C and 160 °C) resulted in the best extraction yield, and therefore were selected for the subsequent trials.

Using the best extraction temperatures and corresponding system pressures, the optimal duration for the hydrothermal extraction process was investigated. The results are summarized in Table 2. The highest purity AX extracts were achieved with an extraction time of 1 h, independent from the temperature and pressure applied. However, with purities ranging between 47–59%, there were generally only small differences regarding the AX content of the four isolates. In contrast, the extraction at 163 °C with a pressure of 6.5 bar was found to be the most efficient in terms of AX-yield, resulting in extraction yields of 23.7% and 25.7% for 1 and 2 h of extraction, respectively. It was further evident that an extraction time of 30 min is not sufficient for a high yield. Using the same temperature and pressure combination for 1 h resulted in a nearly two times higher AX content in the extract. On the other hand, it also appears unnecessary to use extraction times of more than 1 h. The comparison of the products A1 and A3 shows only minor differences in AX yield and extract purity.

It was demonstrated in a number of studies that extraction time is one of the key parameters for both extract yield, and depolymerization, of AX [15,28]. The reason behind this is that water-unextractable AX is covalently and non-covalently linked to cell wall constituents. In order to break these bonds and allow the AX to dissolve, high temperature and pressure can be applied. While this increases the solubility of AX, it creates the dilemma of depolymerizing the AX [29]. As a result, an excessive extraction time (here 2 h) removes more AX from the bran, but also leads to increased degradation of the dissolved AX. Only AX oligo- and polymers were included in the determination of the AX content. Thus, the release of arabinose and xylose monomers due to degradation explains the lower yield and purity, due to longer extraction time. Moreover, the molecular structure and extract purity was compromised by excessive extraction times, as can be seen in comparison of the products A1 and A3. Based on these results, the process parameters to obtain the product A1 were identified as the best hydrothermal extraction.

**Figure 6:**
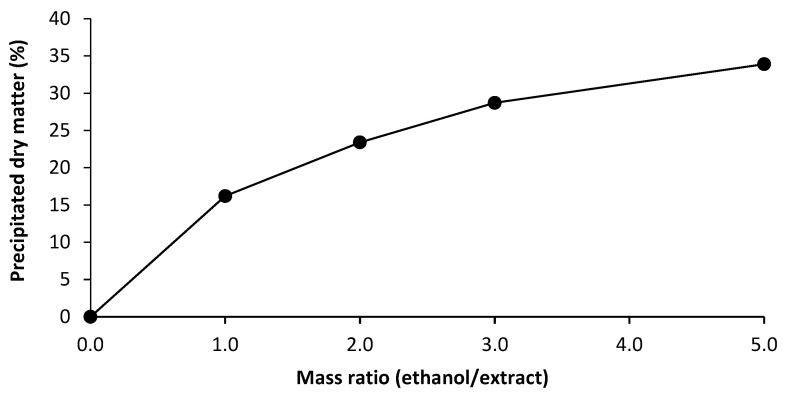
Precipitation of arabinoxylans; amount of precipitated dry matter depending on the mass ratio ethanol/hydrothermal extract.

The final process parameter investigated prior to up-scaling was the precipitation of AX from the extract, using ethanol. While the mass ratio of ethanol/extract used for the results in Table 2 was 5:1, ethanol is also very cost-intensive [31]. The high costs in the use of ethanol are due to its expensive distillation and recovery, as well as the need for explosion-proof equipment. Therefore, the possibility of reducing the amount of ethanol was investigated. This is of particular interest for the possibility of up-scaling the hydrothermal process in further works. The results of the precipitated dry matter as a function of the ethanol/extract mass ratio are shown in Figure 6.

It is evident that generally higher amounts of ethanol result in higher precipitation rates of the extracted dry matter. It is further visible how mass ratios above three lead to a much smaller increase in precipitated dry matter per mass equivalent of ethanol, compared to smaller mass ratios. However, in order to obtain a reasonably high extraction yield, a mass ratio of 5:1 was found to be necessary. This result is generally in good agreement with the existing literature [32].

Overall, the optimized process parameters for hydrothermal extraction of wheat bran AX at laboratory-scale were identified as 1 h extraction at 160 °C, 6.5 bar followed by addition of ethanol equal to five times the extract mass. In addition, to determine the quality of the extraction processes, selected characteristics, beyond the yield and extract purity, were determined and discussed in the following sections. This will help to compare the quality of the hydrothermal extraction with the alkaline/hydrogen peroxide process.

### 3.3. Molecular Mass (MW) of Extracted Arabinoxylans

The MW is among the most important parameters to characterize arabinoxylans. It was demonstrated in a number of studies that the MW has a crucial influence on the physico-chemical, as well as the physiological, properties of AX [30,33].

**Figure 7:**
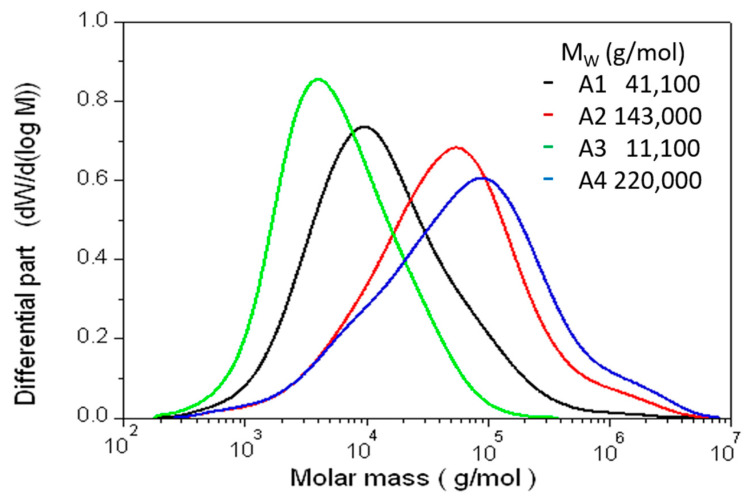
Molecular mass distribution of arabinoxylan products obtained by hydrothermal extraction at laboratory-scale. The four products shown were obtained by the following methods: A1: 1 h, 160 °C, 6.5 bar; A2: 1 h, 150 °C, 4.4 bar; A3: 2 h, 160 °C, 6.5 bar; and A4: 0.5 h, 150 °C, 4.4 bar.

The average MW of the alkaline/hydrogen peroxide extracted sample was determined to 70,200 g/mol (results not shown). In comparison, the results obtained for the hydrothermally extracted products are shown in Figure 7. The average MW of the four products ranged between 11,100 g/mol and 220,000 g/mol. The comparison of the extracts A1 and A3 clearly shows a negative correlation between the extraction time and MW. Although both samples were extracted at 160 °C and 6.5 bar, the product obtained after 1 h had a four times higher molar mass compared to the product after 2 h. A similar trend is visible for the comparison of the products A2 and A4. Furthermore, the comparison of the products A1 and A2 shows that depolymerization of the AX was also dependent on the temperature applied. After 1 h of extraction, the higher temperature resulted in a substantially reduced MW. However, the impact of the extraction time on the MW was found to be much less substantial compared to the temperature applied. It further needs to be mentioned, that the procedures with the highest extract yield and purity induced the most depolymerization.

In order to achieve acceptable yields above 50% by hydrothermal extraction, increased depolymerization needs to be accepted. As a result, the hydrothermally extracted arabinoxylans were found to have a lower MW compared to the alkaline/hydrogen peroxide extract. Similar results were reported previously by Buksa et al. [34]. Since the MW of the extracts obtained at 160 °C and with alkaline/hydrogen peroxide were below 100,000 g/mol, they should be considered as low MW-AX [15]. However, there is evidence for different positive properties for both high, and low, MW-AX [33]. The authors reported higher viscosity and immune-enhancing properties for high MW-AX. On the other hand, AX with MW below 100,000 g/mol enhanced the production of SCFA [33]. In addition, low MW AX was found to inhibit the in vitro starch digestion of AX-rich breads [15]. The product A3 in particular experienced enough depolymerization to result in AX-oligosaccharides (AXOS), rather than polysaccharides. While, traditionally, high MW-AX was considered desirable, some studies have also shown the health-functional properties of AXOS [35,36,37].

Hence, the fractions A1 and A3 remain the most favorable of the hydrothermal extractions, since they can be expected to show valuable physiological health-beneficial properties combined with an efficient extraction.

### 3.4. Arabinose/Xylose (A/X) Ratio of the Extracts

In combination with the MW, the A/X ratio is regarded as an important parameter to characterize AX for both physico-chemical, and functional, properties. The A/X ratio describes the degree of substitution of the xylose backbone with arabinose residues. The A/X ratios of the raw material and the five arabinoxylan extracts are summarized in Table 3.

First, it is remarkable that the highest A/X ratio (0.60) was found in the raw material. All samples obtained by extraction presented a lower degree of substitution. Hence, all extraction processes applied caused a decrease in substitution to varying degrees. Furthermore, the alkaline/hydrogen peroxide process resulted in an A/X ratio determined to 0.42. Thus, resulting in a higher substituted AX compared to the hydrothermal processes. Out of the hydrothermal extractions, products A1 and A4 showed the highest A/X ratios. This result indicates that the extraction time is the most critical factor in terms of de-substitution. Interestingly, higher temperature and pressure resulted in the dissolving of higher substituted AX, as can be seen from the comparison of the products A1 and A2.

Compared to a recent study [25], this indicates a very low substituted AX obtained in this present work, regardless of the extraction procedure. It was reported that highly substituted AX (A/X ratio 0.62) displayed better gelling properties and prebiotic potential compared to lower substituted AX (A/X ratio 0.49) [38]. However, according to the results found by Zhou et al. [39], AX with a lower A/X ratio are more efficient in macrophage phagocytosis and delayed hypersensitivity reaction compared to the enzymatically extracted AX with a higher A/X ratio. Moreover, there appears to be no substantial influence of the A/X ratio on bread and dough properties, when applied in baking experiments [6]. In order to evaluate the health-endorsing properties of the extracted AX more thoroughly, application of the products in vitro and in vivo would be required. Only then could a proper understanding of its potential as a nutraceutical food ingredient be achieved.

### 3.5. Dynamic Viscosity of the Extracted Arabinoxylans

**Figure 8:**
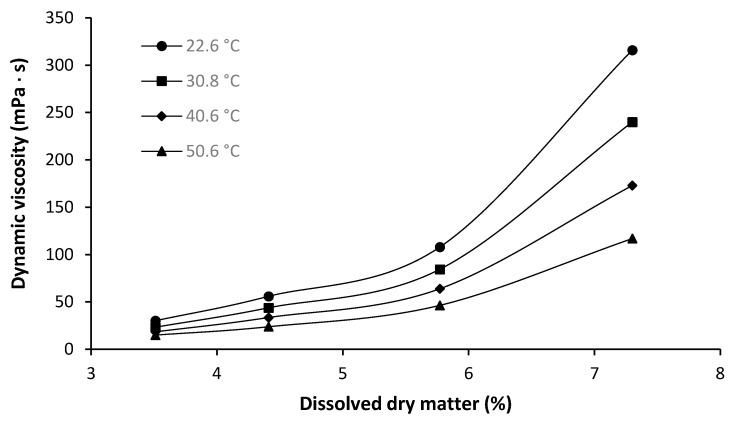
Dynamic viscosity of aqueous solutions of arabinoxylan isolate, obtained by alkaline/hydrogen peroxide extraction, as a function of dry matter. Determination was carried out at a shear rate of 179.6 s^−1^ and temperatures between 22.6 °C and 50.6 °C.

The final important characteristic of AX included in this study is the dynamic viscosity in aqueous solution. It has been established that the health-beneficial effects of AX are related to a number of characteristics, with the viscosity being among the most important [40,41]. Therefore, the dynamic viscosity of the AX extract, obtained by alkaline/hydrogen peroxide extraction, was determined as a function of polymer concentration and temperature (Figure 8). Due to a higher amount of impurities in the hydrothermal extracts, the determination of the dynamic viscosity of these products was heavily compromised (results not shown). As a result, an appropriate evaluation of the data was not possible. However, due to the lower MW of the products A1 and A3, an even higher critical concentration would be expected. Hence, application as a functional food ingredient would be more challenging compared to the alkaline/hydrogen peroxide extract.

As can be seen from Figure 8, the dynamic viscosity decreases as a result of increasing temperature. However, it is remarkable that, with increasing polymer concentration, the effect of changing temperature on the viscosity increases as well. In addition, the dynamic viscosity increases strongly with increasing polymer concentration. It is visible that the relation between viscosity and concentration follows an exponential increase. This increase was clearly much more pronounced at 22.6 °C compared to 50.6 °C.

The exponential increase in dynamic viscosity as a function of polymer concentration, in particular for AX contents in excess of 1%, has been reported by numerous researchers for a variety of AXs from different sources [42,43,44]. The reason behind this was found to be a semi-flexible confirmation of AX, which was shown to be responsible for two different viscosity regimes. These two regimes, a diluted and a concentrated regime, are separated by a single critical concentration [45]. It was further shown, that this critical concentration is in direct correlation with the MW of the AX. As discussed in Section 3.3, the MW of the sample extracted with alkaline/hydrogen peroxide was determined as 70,200 g/mol. As a result the critical concentration, as proposed by Kale, Yadav, Hicks, and Hanah [45], should be found at an approximate AX concentration of 3.4%. Since the obtained extract contained only 69.8% AX, the critical concentration was expected at a dry matter concentration of 4.9%. This expected critical concentration is confirmed by the data presented in Figure 8. It also has to be considered that the 30% of DM which is not AX is expected to have an influence on the experimental result as well. As a result, for the application of this extract, an AX concentration above 4.9% should be applied. Then the formation of a highly viscous gel in the human intestine is possible, increasing the health-beneficial properties.

### 3.6. Comparison of Alkaline/Oxidative with Hydrothermal Extraction

A comparison of the established alkaline/hydrogen peroxide and hydrothermal extraction is shown in Table 4.

The two extraction methods resulted in AX yields of 21.4% and 25.7% of the theoretically extractable arabinoxylan content for the alkaline/oxidative and the hydrothermal process, respectively. It is particularly remarkable, that the hydrothermal laboratory-scale method resulted in a higher yield compared to the established alkaline/hydrogen peroxide method at pilot-scale. Compared to previous reports on the extraction of arabinoxylans, using chemical solution extraction methods, it was comparable to results achieved at lab-scale [39,46]. The few existing pilot-scale studies even reported substantially lower extraction yields, as was summarized in the review of Zhang, Smith, and Li [10]. This is particularly remarkable considering the absence of hazardous chemicals and expensive enzymes. Existing reports on alkaline/hydrogen peroxide extraction often apply barium hydroxide instead of sodium hydroxide, since the Ba^2+^-Ion supports the extraction and separation from beta-glucans [10]. On the downside however, the barium ions require thorough removal before application in food products, while residues of the sodium hydroxide and hydrogen peroxide applied here are unproblematic. Another advantage of the hydrothermal process is the relatively short extraction time of 1 h, compared to 3 h for the alkaline/hydrogen peroxide method.

On the other hand, there are a number of disadvantages for the hydrothermal method. Most noticeable is the lower extract purity, which can compromise the physico-chemical and health-endorsing properties of the AX. This was already evident during the determination of the dynamic viscosity, which was not possible for the hydrothermal extracts. In addition, the higher process temperature and bigger volume of ethanol needed for precipitation, alongside the lower MW and A/X of the final product are further disadvantages of hydrothermal extraction.

Considering all aspects of the extraction process and the products, it was not possible to obtain a hydrothermal extract with similar quality to the alkaline/hydrogen peroxide reference method. Although pilot-scale extractions usually deliver inferior results compared to laboratory-scale, the reference method at pilot-scale was found to remain clearly superior to the hydrothermal method at laboratory-scale. Therefore, no further knowledge could be gained by up-scaling the hydrothermal extraction described here.

## 4. Conclusions

Wheat bran AX were extracted hydrothermally at laboratory-scale and compared to alkaline/hydrogen peroxide extraction at pilot-scale. Since the extract purity, MW, and A/X ratio of the alkaline/hydrogen peroxide extraction were clearly superior to the hydrothermal extraction, this has to remain the standard procedure. Although the extraction conditions of the hydrothermal process were optimized, the resulting product characteristics were inferior to the established method. However, there have been first reports on the combination of hydrothermal extraction with ultrasound or microwaves to obtain better results [30]. If future investigations can apply such techniques in combination with the ideal process conditions identified here, hydrothermal extractions can become a feasible alternative to the established methods. Furthermore, a more thorough understanding of the structure–function relationship of AX should be the aim of future investigations. This could even allow the utilization of heavily degraded AX as a functional food ingredient, when applied appropriately.

## Figures and Tables

**Table 1:** Chemical composition of wheat bran.

Component	Content (% in Dry Matter)
Dry matter (DM) = 86.7%	-
Protein	17.3
Starch	18.5
Minerals	6.1
Lipids	3.4
Total dietary fiber	49.4
Insoluble dietary fiber	47.7
Soluble dietary fiber	1.7
Arabinoxylans	24.5

**Table 2:** Yield and extract purity of different hydrothermal procedures at laboratory-scale.

Product	Temperature (°C)	Pressure (bar)	Extraction Time (h)	Yield (%)	Extract Purity (%)
A1	160	6.5	1	25.7	58.6
A2	150	4.4	1	18.3	56.2
A3	160	6.5	2	23.7	55.4
A4	150	4.4	0.5	9.5	47.3

**Table 3:** Arabinose/xylose ratios of various arabinoxylan extracts.

Extraction Process		Arabinose/Xylose Ratio
Wheat bran		0.60
Alkaline/hydrogen peroxide (pilot-scale)		0.42
Hydrothermal (laboratory-scale)	A1	0.38
	A2	0.32
	A3	0.19
	A4	0.40

**Table 4:** Comparison of alkaline/oxidative and aqueous/hydrothermal extraction procedures for wheat bran arabinoxylans.

Extraction Process	Dimension	Temperature (°C)	Extraction Time (h)	Mass Ratio (Ethanol/Solvent) for Precipitation	Yield (%)	Extract Purity (%)
Alkaline/hydrogen peroxide	Pilot-scale	60	3	3.17	21.4	69.8
Aqueous hydrothermal	Laboratory-scale	160	1	5	25.7	58.6

## Data Availability

Data available on request due to restrictions. The data presented in this study are available on request from the corresponding author. The data are not publicly available due to government restrictions.
